# Non-enveloped virus membrane penetration: New advances leading to new insights

**DOI:** 10.1371/journal.ppat.1010948

**Published:** 2022-12-08

**Authors:** Madison L. Pletan, Billy Tsai

**Affiliations:** 1 Department of Cell and Developmental Biology, University of Michigan Medical School, Ann Arbor, Michigan, United States of America; 2 Cellular and Molecular Biology Program, University of Michigan Medical School, Ann Arbor, Michigan, United States of America; University of Arizona, UNITED STATES

## Abstract

Host cell membranes pose a particular challenge for non-enveloped viruses. Whereas enveloped viruses enter cells by fusing their lipid envelopes with the cellular membrane, non-enveloped viruses generally must (1) enter cells via endocytosis, then (2) penetrate the cellular endomembrane to reach the cytosol. Only then can the viruses begin to replicate (or transit to the nucleus to replicate). Although membrane penetration of non-enveloped viruses is a crucial entry step, many of the precise molecular details of this process remain unclear. Recent findings have begun to untangle the various mechanisms by which non-enveloped viral proteins disrupt and penetrate cellular endomembranes. Specifically, high-resolution microscopy studies have revealed precise conformational changes in viral proteins that enable penetration, while biochemical studies have identified key host proteins that promote viral penetration and transport. This brief article summarizes new discoveries in the membrane penetration process for three of the most intensely studied families of non-enveloped viruses: reoviruses, papillomaviruses, and polyomaviruses.

## A. Structural shifts allow membrane penetration: Bluetongue virus and rhesus rotavirus

Distinct conformational changes experienced by viral capsid proteins during entry allow the viral particles to properly penetrate host endomembranes before productive infection. For viruses that penetrate the endosomal membrane, conformational shifts are often triggered by the low pH of endosomal acidification [1]. Two recent papers applied a cryo-electron microscopy (cryo-EM) approach to illuminate structural rearrangements imparted on reovirus that promote its membrane penetration.

**Fig 1:**
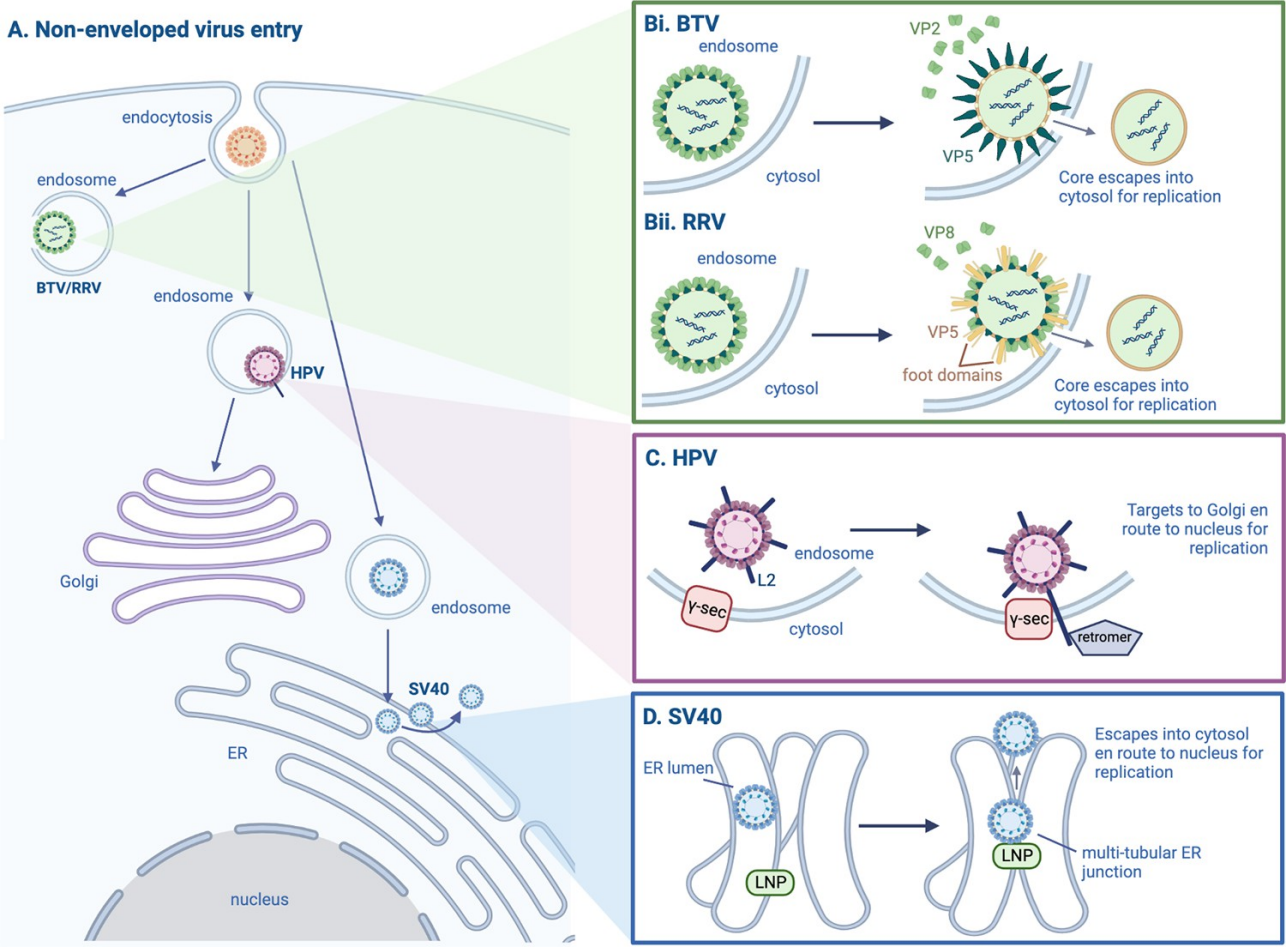
Mechanisms of non-enveloped virus membrane penetration. (**A**) After uptake by endocytosis, BTV, RRV, HPV, and SV40 undergo membrane penetration at different locations in the cell. (**B**) For reoviruses BTV and RRV, endosomal acidification triggers conformational changes in and/or dissociation of indicated capsid proteins, leading to perforation and rupture of the endosomal membrane. (**C**) The host protein γ-secretase drives insertion of the HPV capsid protein L2 across the endosomal membrane, where its inserted state is stabilized by the host protein retromer. (**D**) SV40 induces the formation of multi-tubular ER junctions, stabilized by the host protein Lunapark, where the viral particles can penetrate the membrane and escape into the cytosol. Created with BioRender.com. BTV, bluetongue virus; ER, endoplasmic reticulum; HPV, human papillomavirus; LNP, Lunapark; RRV, rhesus rotavirus; SV40, simian virus 40.

Structurally, reoviruses consist of segmented double-stranded RNA genomes packaged in layered, concentric protein capsids [2]. The basic reovirus entry pathway has several key steps (Fig 1A). First, the virus is taken up by endocytosis; second, acidification in the late endosome induces viral penetration of the endosomal membrane; third, the viral core escapes into the cytosol for replication [2,3]. Reoviruses can infect a wide range of hosts: Bluetongue virus (BTV) is an endemic pathogen of sheep and other livestock, while rhesus rotavirus (RRV) is one of many rotaviruses that collectively represent the most common cause of severe childhood diarrhea [4,5].

To examine the conformational changes experienced by BTV proteins during endosomal acidification, Xia and colleagues captured cryo-EM images of purified BTV particles upon exposure to low pH [4]. Three-dimensional reconstruction of the BTV particle revealed several distinct structural rearrangements at low pH (Fig 1Bi): (1) dramatic unfolding of the membrane-penetrating protein VP5; (2) subtle shift of the capsid protein VP7; and (3) dissociation of the receptor-binding protein VP2. The authors further demonstrated that a cluster of histidine residues in the VP5 “dagger domain” act as a pH sensor, and protonation of these residues allows the protein to unfurl and form a long stalk. Next, the authors used cryo-electron tomography (cryo-ET) to examine the interaction of purified BTV particles with target membranes—in this case, liposomes. After a low-pH wash, cryo-ET images showed the tips of VP5 stalks interacting with target membranes. Furthermore, the interaction between a virus and a liposome appeared to create a single perforation at the interaction site.

Using a similar strategy, Herrmann and colleagues also took advantage of cryo-EM techniques to elucidate a critical step in RRV entry [5]. After endocytosis, the spike protein VP4 must be cleaved to VP5 and VP8, which rearrange into a “reversed” conformation and execute membrane penetration [6]. What does the rearrangement entail? By solving the three-dimensional structure of re-coated RRV particles, the authors showed that the capsid proteins can spontaneously switch to the reversed conformation on the virion surface. In this conformation, the VP5 hydrophobic loops protrude to capture a target membrane, the previously buried “foot domain” of VP5 is unfolded to project outwards, and VP8 gradually dissociates from the particle (Fig 1Bii). To identify a potential intermediate folding state, the authors generated a mutant spike protein in which the folded foot domain is stabilized by disulfide bonds. Virus particles with this mutation displayed protruding hydrophobic loops but not unfolded foot domains—this may represent a possible intermediate conformation. Cryo-ET of RRV-infected cells, although not captured with a sufficient resolution to reveal the precise virus–membrane contacts, suggested that the virus moves close enough to the target membrane for the projected foot domain to embed. This was consistent with previously published cryo-ET studies of rotavirus entry [7].

Taken together, Herrmann and colleagues and Xia and colleagues provide the highest-resolution picture of membrane penetration by reoviruses—indeed, of all non-enveloped viruses—available to date [2]. Since other reovirus family members, such as orthoreoviruses, have long been known to undergo similar outer capsid conformational shifts prior to penetration of the endosomal membrane and escape of the viral core [8,9], these findings may illuminate their entry mechanisms as well.

## B. Host proteins promote membrane penetration: Human papillomavirus

The reoviruses described above are capable of sensing low pH, shifting their capsid protein conformations, and perforating target membranes largely unaided. However, many non-enveloped viruses also depend on host proteins to assist in the membrane penetration step. One notable example is the human papillomavirus (HPV). Several publications in recent years have highlighted the importance of host factors in facilitating HPV membrane penetration.

As the most common sexually transmitted infection in the United States, HPV causes a range of cervical, oropharyngeal, and anogenital cancers [10]. Structurally, an HPV particle consists of a double-stranded DNA genome packaged in an icosahedral capsid, which is composed of L1 and L2 proteins [11]. During entry (Fig 1A), the virus is taken up by endocytosis and partially disassembles in the endosome, where it then penetrates the endosomal membrane. However, unlike the reoviruses, the HPV particle does not rupture the endosomal compartment or escape into the cytosol; rather, it simply inserts its L2 capsid protein across the host membrane [10,12,13]. Functionally, membrane insertion of L2 exposes this viral protein to the cytoplasm, which in turn recruits the host protein retromer that sorts the virus to the Golgi. HPV then remains in the Golgi until mitosis, when following Golgi fragmentation and nuclear envelope breakdown, Golgi-derived vesicles harboring the virus traffic to the nucleus [10,12]. Because these vesicles are thought to remain intact until the end of mitosis, it is unclear how the viral genome ultimately escapes into the nucleoplasm to continue its replication process [14].

How does viral L2 insert across the endosomal membrane? Recent publications have shed light on two important elements in the insertion process: (1) specific regions in the L2 sequence; and (2) interaction with host proteins. Regarding the L2 protein itself, Zhang and colleagues demonstrated that HPV16 L2 contains a short basic sequence (RKRRKR) at its C-terminus that acts as a cell-penetrating peptide (CPP) [15]. As shown using proximity ligation and split-GFP assays, although virions with mutations in the CPP sequence were able to attach to and enter cells, they were trapped in endosomes and could not penetrate the endosomal membrane. On the host–factor side of the equation, Inoue and colleagues used immunoprecipitation and alkali extraction assays to demonstrate that the transmembrane host protein γ-secretase binds to L2, driving insertion of L2 across the host membrane (Fig 1C) [16]. Disruptions in this process—including knockdown or inhibition of γ-secretase, or depletion of the γ-secretase adaptor protein p120—all prevented L2 membrane penetration and blocked productive infection [16,17]. While Inoue and colleagues identified γ-secretase as a novel, non-classical chaperone for HPV, the precise molecular details of membrane penetration remained unclear. Most recently, Xie and colleagues demonstrated that the cytosolic sorting factor retromer also plays a role in L2 insertion: Depletion of retromer significantly reduced the amount of membrane-inserted L2 [18]. After performing time-course experiments, the authors posited that L2 dynamically and transiently penetrates the endosomal membrane (aided by γ-secretase and the CPP) but requires binding to retromer to stabilize the membrane-inserted state (Fig 1C).

In sum, studies of HPV–host interactions have revealed novel roles for well-known proteins like γ-secretase and retromer in promoting and stabilizing HPV membrane penetration. These studies highlight the usefulness of viruses as tools to gain fresh insights into basic cell biology. Further work is needed to elucidate the molecular basis for sustained L2 insertion, as well as the process by which HPV ultimately egresses from the Golgi-derived vesicles to complete its replication cycle.

## C. Membrane penetration requires a virus-induced membrane structure: Simian virus 40

Both reoviruses and papillomaviruses penetrate the endosomal membrane, but polyomavirus bides its time, waiting to penetrate a host membrane until it arrives at the endoplasmic reticulum (ER). This is exemplified by the prototype polyomavirus simian virus 40 (SV40), whose membrane penetration involves formation of a virus-induced sub-organellular structure in the ER, a process that requires the action of many host ER factors.

Polyomaviruses are small, double-stranded DNA tumor viruses encapsulated in icosahedral capsids [19,20]. While polyomaviruses can cause severe human diseases and cancers, especially in immunocompromised patients, the best-studied polyomavirus historically is the monkey virus SV40 [10]. SV40’s entry pathway uniquely relies on the ER compartment (Fig 1A): After uptake by endocytosis, the virus is first delivered to the endosome and then sorted to the ER, where it penetrates the ER membrane at specific sites termed “foci” in order to reach the cytosol [20,21]. Upon reaching the cytosol, the virus is partially disassembled and traffics to the nucleus for replication [10,20].

In order to penetrate the ER, SV40 remodels the ER membrane to facilitate membrane penetration, recruiting a suite of transmembrane proteins, molecular motors, and cytosolic factors to extract virions into the cytosol [22]. Though nearly a dozen individual host components have been identified as indispensable for the penetration/extraction process [21,22], the molecular architecture of the SV40-induced ER foci structure remained mysterious until recently. To visualize the foci more directly, Bagchi and colleagues used focused ion beam scanning electron microscopy (FIB-SEM) to reconstruct three-dimensional renditions of SV40 penetrating the ER (Fig 1D) [23]. These images reveal SV40 escaping the ER from the center of flower-like, multi-tubular ER junctions. Lending further support to this finding, the authors identified Lunapark, an ER membrane protein that stabilizes typical three-way junctions of the web-like reticular ER, as required for both foci formation and for viral penetration [23,24]. In this model, the authors posit that SV40 recruits host proteins to reorganize the ER into multi-tubular junctions, where the membrane is most vulnerable and therefore ideally suited for viral penetration.

This study provides a detailed picture of the extent to which SV40 can rearrange not just individual host proteins but entire regions of the ER structure during its infection process. Thus, not only are viruses capable of shifting their own conformations (BTV and RRV) and recruiting host proteins (HPV), but they may also substantially reorganize organelle architecture to facilitate penetration (SV40).

## D. Future directions

In this brief article, we reviewed exciting recent developments elucidating the membrane penetration process for reoviruses, papillomaviruses, and polyomaviruses. While these non-enveloped viruses share some commonalities in their entry pathways, their membrane penetration steps are distinct in the precise sub-cellular location (endosome versus ER) and whether host factors are involved. Of particular interest are the advances in understanding non-enveloped penetration via innovative imaging techniques. Application of cryo-EM, as used successfully with reoviruses, to other non-enveloped viruses has the promise to further illuminate the mysterious molecular details of the membrane penetration process.
